# Dynamics of uterine microbiota in postpartum dairy cows with clinical or subclinical endometritis

**DOI:** 10.1038/s41598-020-69317-z

**Published:** 2020-07-23

**Authors:** O. Bogado Pascottini, S. J. Van Schyndel, J. F. W . Spricigo, J. Rousseau, J. S. Weese, S. J. LeBlanc

**Affiliations:** 1 Population Medicine, Ontario Veterinary College, University of Guelph, Guelph, ON N1G 2W1 Canada; 2 Department of Animal Biosciences, University of Guelph, Guelph, ON N1G 2W1 Canada; 3 Department of Pathobiology, Ontario Veterinary College, University of Guelph, Guelph, ON N1G 2W1 Canada

**Keywords:** Bacteriology, Urogenital reproductive disorders, Reproductive signs and symptoms

## Abstract

Our objectives were to describe and compare the uterine bacterial composition of postpartum Holstein cows diagnosed as healthy (n = 8), subclinical endometritis (SCE; n = 8), or clinical endometritis (CE; n = 5) in the fifth week postpartum. We did metagenomic analyses of 16S rRNA gene sequences from endometrial cytobrush samples at 10, 21, and 35 days in milk (DIM), and endometrial bacterial culture at 35 DIM. Uterine bacterial composition in healthy, SCE, and CE was stable at 10, 21, and 35 DIM. Alpha and beta diversities showed a different uterine microbiome from CE compared to healthy or SCE, but no differences were found between healthy and SCE cows. At the phylum level, the relative abundance of *Bacteroidetes* and *Fusobacteria*, and at genera level, of *Trueperella* was greater in CE than healthy or SCE cows. *Trueperella pyogenes* was the predominant bacteria cultured in cows with CE, and a wide variety of bacterial growth was found in healthy and SCE cows. Bacteria that grew in culture were represented within the most abundant bacterial genera based on metagenomic sequencing. The uterine microbiota was similar between SCE and healthy, but the microbiome in cows with CE had a loss of bacterial diversity.

## Introduction

Multiple potential mechanisms that drive reproductive tract inflammatory disease in dairy cattle have been discovered^1,2^, but questions remain about the nature and causes of the different manifestations of reproductive tract infection and inflammation. Remarkable changes in the composition of the microbiome occur in postpartum cows that develop uterine disease^3,4^, but few papers have described the dynamics of the uterine microflora in healthy postpartum cows^5,6^. A robust but well-regulated postpartum uterine inflammatory response is necessary to avoid disease^7^. However, in early lactation, cows experience a state of immune dysfunction concurrent with changes in metabolism associated with physiologic adaptations to support milk production. Although certain phyla and genera of bacteria are reasonably consistently associated with some forms of uterine disease, the changes of the microbiome and the host interaction with it in the progression or avoidance of disease are only partially understood. Metritis (overt systemic illness due to uterine infection, characterized by fetid vaginal discharge) and purulent vaginal discharge (PVD) without systemic signs have commonly been associated with *Trueperella pyogenes* using culture-dependent methods^8,9,10^. Clinical endometritis (CE) is PVD with concurrent endometritis based on > 5% polymorphonuclear (PMN) neutrophils^7,11^. However, with the advent of culture-independent methods using metagenomic sequencing, it has been shown that loss of diversity and increase in abundance of *Bacteroides*, *Porphyromonas*, and *Fusobacterium* were associated with metritis and PVD in postpartum cows^3,4,12^.

Numerous studies attempted to associate the presence of bacteria with subclinical endometritis (SCE; absence of PVD and > 5% endometrial PMN), but the role of pathogenic bacteria in SCE remains unclear^13^. Using culture-dependent methods Sens and Heuwieser^14^ showed that *α-hemolytic Streptococci* or *Trueperella pyogenes* in the second week postpartum were associated with SCE diagnosed in the following two weeks. Similarly, Prunner et al*.*^15^ demonstrated that cows diagnosed SCE at 28 days in milk (DIM) had greater bacterial growth density in the second week postpartum than healthy cows. Other studies did not find associations between bacterial culture results and SCE at the time of diagnosis^15,16^. Given that only approximately 1% of bacteria are culturable^6^, the study of the 16S rRNA gene will potentially allow description of the full uterine microbiome signature in cows with SCE.

Wang et al*.*^17^ investigated the bovine uterine microbiota in uterine flush samples collected at 30 DIM by high throughput sequencing of 16S rRNA. They concluded that SCE was not associated with the uterine pathogens that they found for CE in the same study (increased abundance of *Fusobacterium* and unique presence of *Trueperella* and *Peptoniphilus*). Evaluating the uterine microbiota at the time of diagnosis is a good first step. However, it will be more informative to investigate the changes in the uterine microbiota before and at diagnosis of SCE, and to compare it with the microbiome composition in healthy and CE cows. Investigation of microbiome dynamics before the diagnosis of PVD has been reported, but samples were collected from the vagina^18^. For this study, we hypothesized that the dynamics of uterine microbiota would differ among cows that were healthy or had SCE or CE in the fifth week postpartum. The objectives were to describe and compare the uterine bacterial composition of postpartum dairy cows diagnosed as healthy, with SCE, or CE in the fifth week postpartum in samples collected at 10, 21, and 35 DIM. We also aimed to compare the uterine bacteria recovered in culture with the metagenomic profile in samples collected at 35 DIM.

## Materials and methods

### Animals and management

Cows for this study come from a larger experiment which evaluated the metabolic effects of postpartum anti-inflammatory treatment (meloxicam) in clinically healthy dairy cows. In the underlying study, 20 out of 42 Holstein cows received subcutaneous injections of meloxicam (0.5 mg/kg of body weight; Metacam, Boehringer Ingelheim Canada Ltd., Burlington, ON, Canada) once daily on 10, 11, 12 and 13 DIM. For the present study, 21 cows were retrospectively and deliberately selected to balance as nearly as possible for meloxicam treatment (n = 9 were treated with meloxicam and n = 12 were non-treated control), parity, and uterine health status (described below). Meloxicam treatment did not affect the uterine inflammatory status^19^ nor the uterine microbiome composition^20^. This lack of effect allowed us to study the associations between reproductive tract inflammatory disease and the uterine microbiome in the postpartum period.

Cows were managed according to the guidelines set by the National Farm Animal Care Council. Animal handling procedures and sampling were approved by the University of Guelph Animal Care Committee (Animal Utilization Protocol 3852). Sampling was conducted from April to August 2018 at the University of Guelph Livestock Research and Innovation Centre, Dairy Facility (Elora, ON, Canada). Briefly, pregnant dry cows were housed in free-stall barns and moved to individual calving pens when showing signs of imminent calving (e.g., swelling of the vulva and relaxation of the pelvic ligaments) or 48 h before expected calving. Five days after calving, cows were moved to a free-stall lactating pen, where they remained until the end of the experiment (35 DIM). Cows were fed for ad libitum intake and diet data are reported in Pascottini et al*.*^19^. Milking was done twice daily in a rotary parlour. Only cows considered clinically healthy having unassisted calving, and no retained placenta, metritis, or other clinical disease before or during the study period were included in the present analysis. None of the cows were treated with antibiotics from calving to 35 DIM (locally or systemically).

### Sampling and case definition

Endometrial cytobrush samples were collected at 10, 21, and 35 DIM. After cleansing the perineal area of the cow with water and soap, 70% isopropyl alcohol was sprayed, and dried using paper towel. A sterile cytobrush rod (covered with a sterile sanitary sheath) was introduced into the vagina and guided through the cervix per rectum. Once the tip of the rod reached the uterine body, the sanitary sheath was pulled back, the cytobrush was exposed from the rod, and rotated against the dorsal wall of the uterine body with gentle pressure of the index finger through the rectum. The cytobrush was retracted into the rod and removed from the vagina. Once outside the genital tract, the cytobrush was gently rolled onto a sterilized microscope slide. The head of the cytobrush was then cut with sterile scissors, placed in a sterile 2 mL cryovial, and stored at − 80 °C within 15 min. Before this last step, in samples collected at 35 DIM, a sterile swab was gently rolled against the cytobrush bristles. Swabs were then transferred to a tube containing anaerobic transport medium (BBL Culture Swab, BD, Mississauga, ON, Canada), placed on ice, and processed within 2 h of collection. After each cytobrush sampling, vaginal discharge was evaluated using the Metricheck device (Simcrotech, Hamilton, New Zealand) and scored as 0 = clear mucus, 1 = mucus with flecks of pus, 2 = mucopurulent discharge (≤ 50% pus), and 3 = purulent discharge (> 50% pus). Cytology slides were stained using May-Grunwald-Giemsa stain, 300 cells were counted per slide in multiple fields, and the PMN to epithelial cell proportion was assessed.

Reproductive tract inflammatory condition was classified at 35 DIM as healthy (n = 8), SCE (n = 8) or CE (n = 5). The criteria for SCE were absence of mucopurulent vaginal discharge (Metricheck score < 2) but with ˃ 5% endometrial PMN^11^. There is inconsistency in case definitions of PVD and CE. Many cows with PVD do not have concurrent endometritis based on cytology^11^. Here we use the term CE as precisely as possible, so the criteria for CE were mucopurulent or purulent vaginal discharge (Metricheck score ≥ 2) with ˃ 5% endometrial PMN. Healthy cows had neither SCE nor CE (Metricheck score < 2 with ≤ 5% endometrial PMN).

### Aerobic and anaerobic bacterial culture

Endometrial swabs were submitted to Animal Health Laboratory, University of Guelph for aerobic and anaerobic bacterial culture. Briefly, for aerobic culture, swabs were plated on blood agar (BA) and MacConkey agar (MAC) plates and incubated at 35 °C either in presence of 5% CO_2_ (for BA) or in atmospheric air (for MAC). For anaerobic culture, the samples were plated on Brucella agar and phenylethyl alcohol agar plates and incubated at 37 °C. Aerobic culture plates were checked for the presence of bacterial growth at 24 and 48 h whereas anaerobic culture plates were checked at 48 h. All morphologically different bacterial colonies were identified using Matrix Assisted Laser Desorption-Ionization Time-of-Flight Mass Spectrometry (MALDI-TOF MS, Bruker Scientific LLC) by directly transferring individual colonies onto a stainless steel target plate and overlying them with 1 µl of α-cyano-4-hydroxycinnamic acid (HCCA) matrix. Generated spectra were compared to the spectra present in the research use only (RUO) library (Compass 4.1 with 8468 spectra present, Bruker Scientific). The bacterial identification was reported to the species level if the MALDI-TOF score was above 2.00 or to the genus level if the score was between 1.7 and 1.9.

### DNA extraction and 16S rRNA gene amplification and sequencing

DNA was extracted from each cytobrush sample using the QIAamp Microbiome kit, following the manufacturer’s protocol (Qiagen Inc.; Toronto, ON, Canada). DNA concentration was measured by a NanoDrop ND-1000 UV–Vis spectrophotometer (Thermo Fisher Scientific; Wilmington DE, USA) based on the absorbance at 260 nm and using the Beer-Lambert equation. The DNA quality was assessed by using the ratio of 260/280 nm. The average 260/280 ratio was 1.93 with a range of 1.5 to 2.2. To assess the bacterial microbiota, PCR amplification of the V4 hypervariable region of the 16S rRNA gene was performed using the forward primer 515F-modand reverse primer806R-mod^21^. The forward and reverse primers were designed to contain an Illumina overhang adapter sequence (Illumina; San Diego, CA, USA) in order to anneal them to primers containing the Illumina adaptors plus the 8 bp identifier indices. The following PCR conditions were used: 3 min at 94 °C for denaturing, followed by 30 cycles of 45 s at 94 °C, 60 s at 52 °C, and 60 s at 72 °C, with a final elongation of 10 min at 72 °C. After amplification, the PCR products were evaluated by electrophoresis in 1.5% agarose gel to ensure amplification had occurred and was the expected size of approximately 400 bp. The PCR product was purified with the Mag-Bind Rxn Pure Plus kit (Omega Bio-Tek, CA, Norcross, GA, USA) by mixing 20 μL of amplicon with 25 μL of Mag-Bind in a 96 well flat-bottom micro-titer plate. After incubation for 5 min at room temperature, the beads were separated and washed twice with 80% ethanol and then eluted in 32 μL of 10 mM Tris pH 8.5 buffer solution. A second PCR was performed to attach dual indices and Illumina sequencing adapters using the Nextera XT Index kit (Illumina). The conditions of this PCR included: 3 min at 94 °C followed by 8 cycles of 30 s at 94 °C, 30 s at 55 °C, and 30 s at 72 °C, plus a final step of 10 min at 72 °C. After purification of these amplicons, the samples were quantified by spectrophotometry using the NanoDrop. Normalization was performed with a Nextera XT DNA library prep kit by the Agriculture and Food Laboratory (AFL), University of Guelph. The AFL performed sequencing of the library pool using an Illumina MiSeq Reagent kit with a 2 × 250 bp read length.

### Statistical analyses

**Figure 1:**
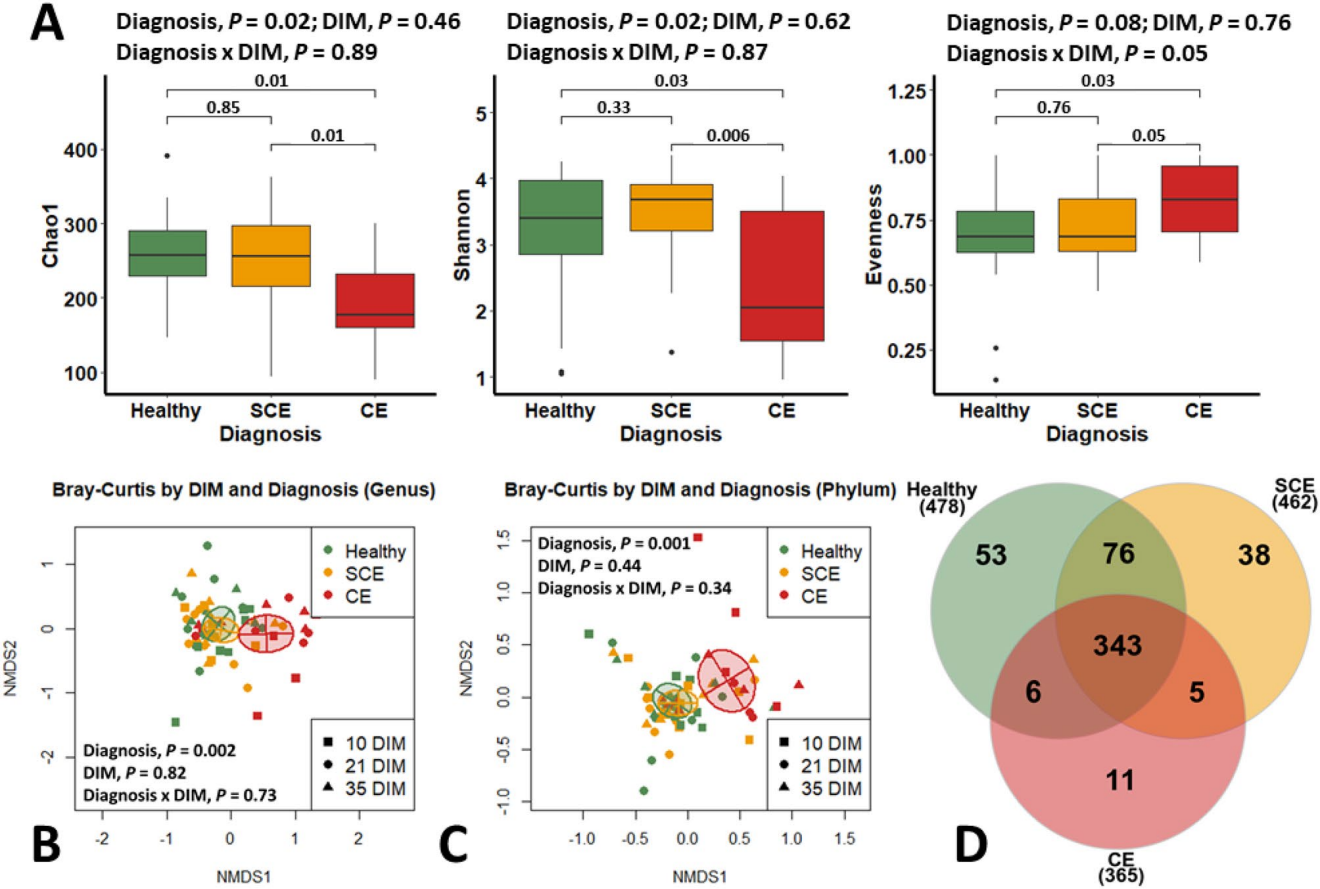
Uterine bacterial composition of postpartum dairy cows (n = 21) in samples collected at 10, 21, and 35 days in milk (DIM). Cows were retrospectively selected based on their uterine health status in the fifth week postpartum and classified as healthy (n = 8), with subclinical endometritis (SCE; n = 8; < 50% purulent vaginal discharge and > 5% endometrial neutrophils (PMN)), or with clinical endometritis (CE; n = 5; ˃ 50% purulent vaginal discharge and > 5% endometrial PMN). (**A**) Alpha diversity for bacterial genera, (**B**, **C**) beta diversity (principal coordinate analysis (Bray–Curtis) for bacteria genera and phyla, and (**D**) number of bacteria core genera (Venn diagram) were similar between healthy and SCE but different for cows with CE.

**Figure 2:**
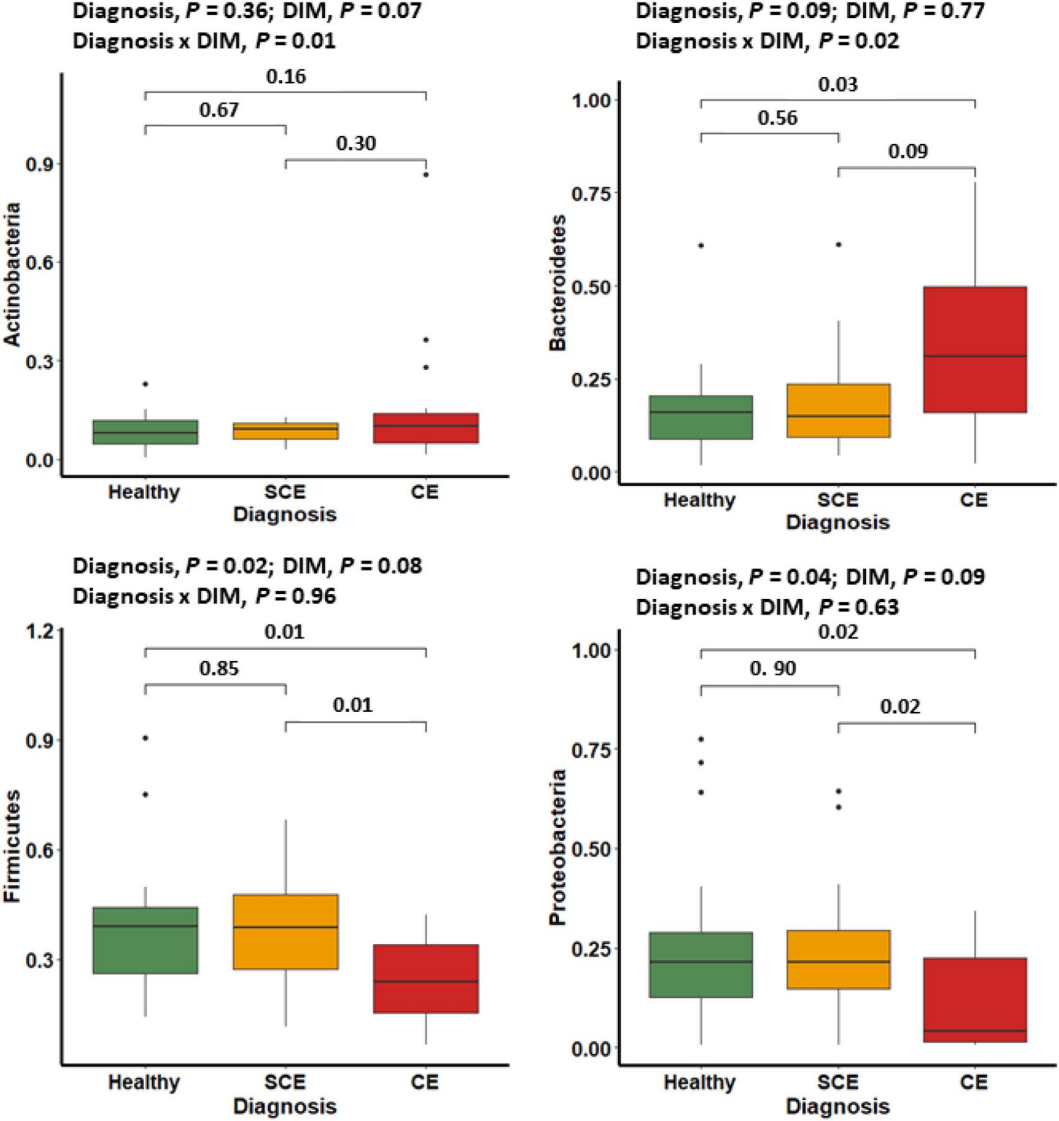
Relative abundance of the most influential bacterial phyla in postpartum dairy cows (n = 21) in samples collected at 10, 21, and 35 days in milk (DIM). Cows were retrospectively selected based on their uterine health status in the fifth week postpartum and classified as healthy (n = 8), with subclinical endometritis (SCE; n = 8; < 50% purulent vaginal discharge and > 5% endometrial neutrophils (PMN)), or with clinical endometritis (CE; n = 5; ˃ 50% purulent vaginal discharge and > 5% endometrial PMN). No differences in relative abundance in bacteria phyla were found between healthy and SCE cows (*P* ˃ 0.56). Cows with CE had lesser relative abundance of *Firmicutes* and *Proteobacteria* than healthy or SCE cows (*P* < 0.03). Cows with CE had greater relative abundance of *Bacteroidetes* than healthy cows (*P* = 0.03).

Downstream analyses were carried out in RStudio (version 3.6.3; R Core Team, Vienna, Austria) using the packages vegan^22^, fossil^23^, and phyloseq^24^, unless otherwise stated. In a first step, decontamination of the endometrial samples was performed using the decontam R package^25^ using the sequencing of 6 sterile cytobrushes exposed to the environment (in the air for 5 s) of the barn in which uterine samples were collected (Supplemental Figure S1). For all analyses, the cow was considered as the unit of interest. For beta diversity (phylum and genera levels), principal coordinate analysis (Bray–Curtis) was used to assess differences in uterine bacterial composition by reproductive tract inflammatory disease condition and DIM at sampling, and their outcomes evaluated using non-parametric multivariate analysis of variance (PERMANOVA) accounting for repeated measures. Venn diagrams^26^ were generated to show the number of core bacterial genera (having ˃ 1% abundance) in healthy, SCE, and CE cows. The *simper* function (package vegan) was used to set the similarity percentages and to extract the most influential bacteria (phylum and genera levels) to compare their relative abundance. Alpha diversity indices for bacterial genera (Chao1, Shannon-Weiner, and Camargo’s evenness; reported in Fig. 1A), and Phylum and Genera-level data (reported in Figs. 2 and 4, respectively) were analyzed with mixed linear regression models in SAS (version 9.4; SAS Institute). Because relative abundance and Evenness data could vary between 0 and 1 and were not normally distributed, the raw data were logit-transformed for analysis, with a small bias correction factor for zero values. Repeated measures were accounted for using an autoregressive type 1 covariance structure, and denominator degrees of freedom were calculated with the Kenward-Rogers 2 adjustment. All models included the effects of diagnosis (numerator degrees of freedom (df) = 2; denominator df = 17 to 24), DIM (numerator df = 2; denominator df = 34 to 37), and the interaction of diagnosis and DIM (numerator df = 4; denominator df = 35 to 39). Linear discriminant analysis effect size (LDA-LEfSe) was used to describe both the statistical significance and biological relevance among healthy, SCE, and healthy cows at the phylum and genera levels. The LDA-LEfSe was performed using the online Galaxy interface (https://huttenhower.sph.harvard.edu/galaxy/)27 with uterine health status as the main class, DIM at sampling as the subclass, and the cow as the subject, using an alpha of 0.05 and an effect size threshold of 3.5. Heatmaps were built (package Heatplus^28^) to illustrate bacteria phyla and genera fold changes (average linkage clustering based on Bray–Curtis distance) and similarity dendrograms (based on Bray–Curtis distance and unweighted pair group method with arithmetic mean clustering) in healthy, SCE, and CE cows by DIM at sampling. Network analysis plots based on Spearman’s correlations were constructed (package qgraph^29^) to illustrate the associations by relative abundance among the most influential bacteria genera in healthy, SCE, and CE cows.

## Results

### Descriptive statistics

The experimental groups consisted of cows classified as healthy [n = 8; parity 1.5 ± 0.5 (mean ± SD) and BCS 3.8 ± 0.2], SCE (n = 8; parity 1.6 ± 0.5 and BCS 3.7 ± 0.3), or CE (n = 5; parity 1.4 ± 0.5 and BCS 3.8 ± 0.2). The vaginal discharge score at 35 DIM was 0.2 ± 0.5, 0.1 ± 0.4, and 2.4 ± 0.6 for healthy, SCE, and CE cows, respectively. The endometrial PMN% at 35 DIM was 1.1 ± 0.9, 24.7 ± 11.3, 27.2 ± 14.8 for healthy, SCE, and CE cows, respectively. Information regarding reproductive tract inflammatory scores and bacterial culture results at the individual cow level is shown in Supplementary Table S1.

### Alpha and beta diversities

**Figure 3:**
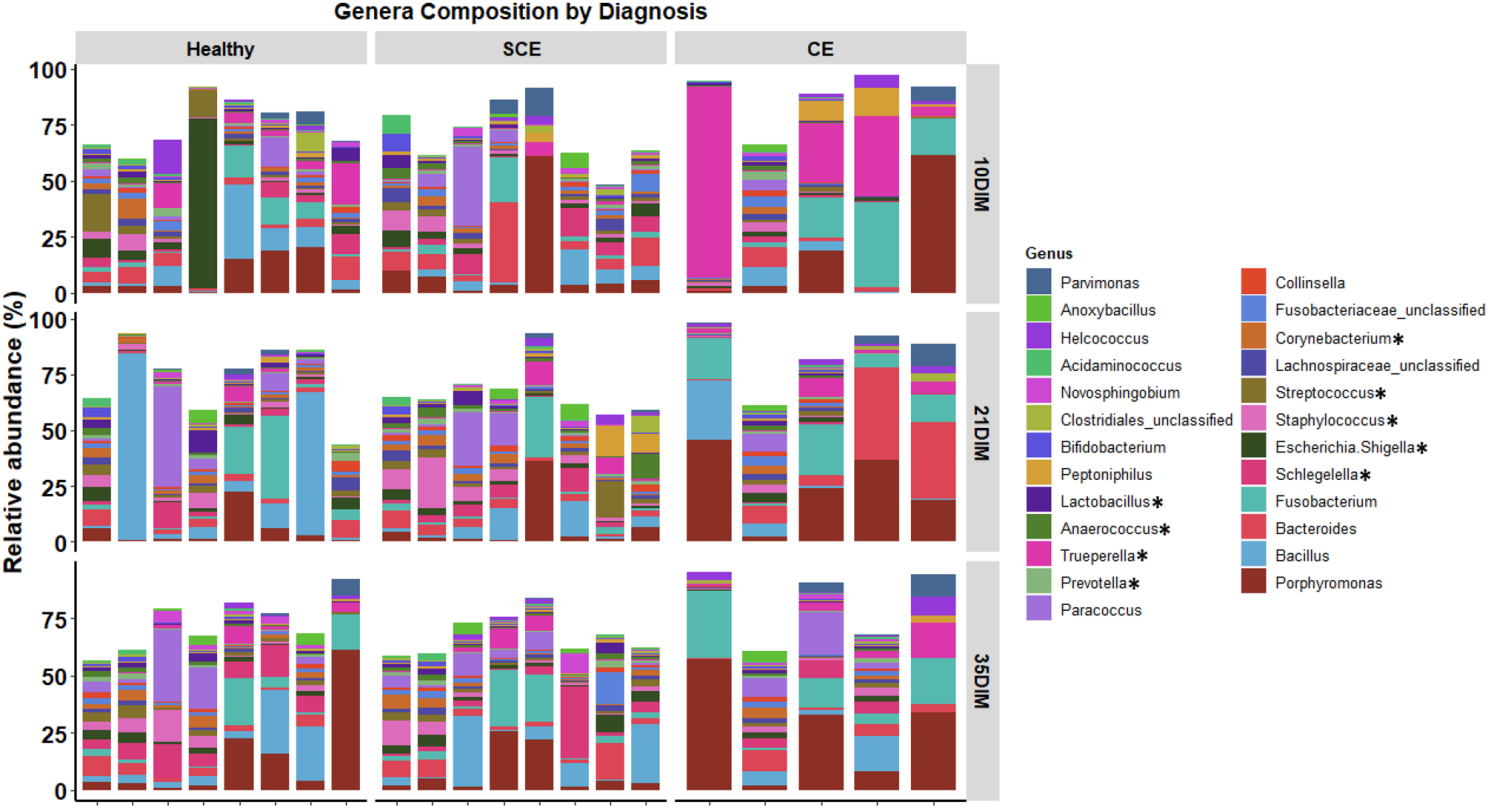
Relative abundance of the most influential bacterial genera of postpartum dairy cows (n = 21) in samples collected at 10, 21, and 35 days in milk (DIM). Cows were retrospectively selected based on their uterine health status in the fifth week postpartum and classified as healthy (n = 8), with subclinical endometritis (SCE; n = 8; < 50% purulent vaginal discharge and > 5% endometrial neutrophils (PMN)), or with clinical endometritis (CE; n = 5; ˃ 50% purulent vaginal discharge and > 5% endometrial PMN). No differences in relative abundance was found between healthy cows and SCE (*P* > 0.05). Asterisks represent differences between CE and healthy cows or SCE (*P* < 0.05).

**Figure 4:**
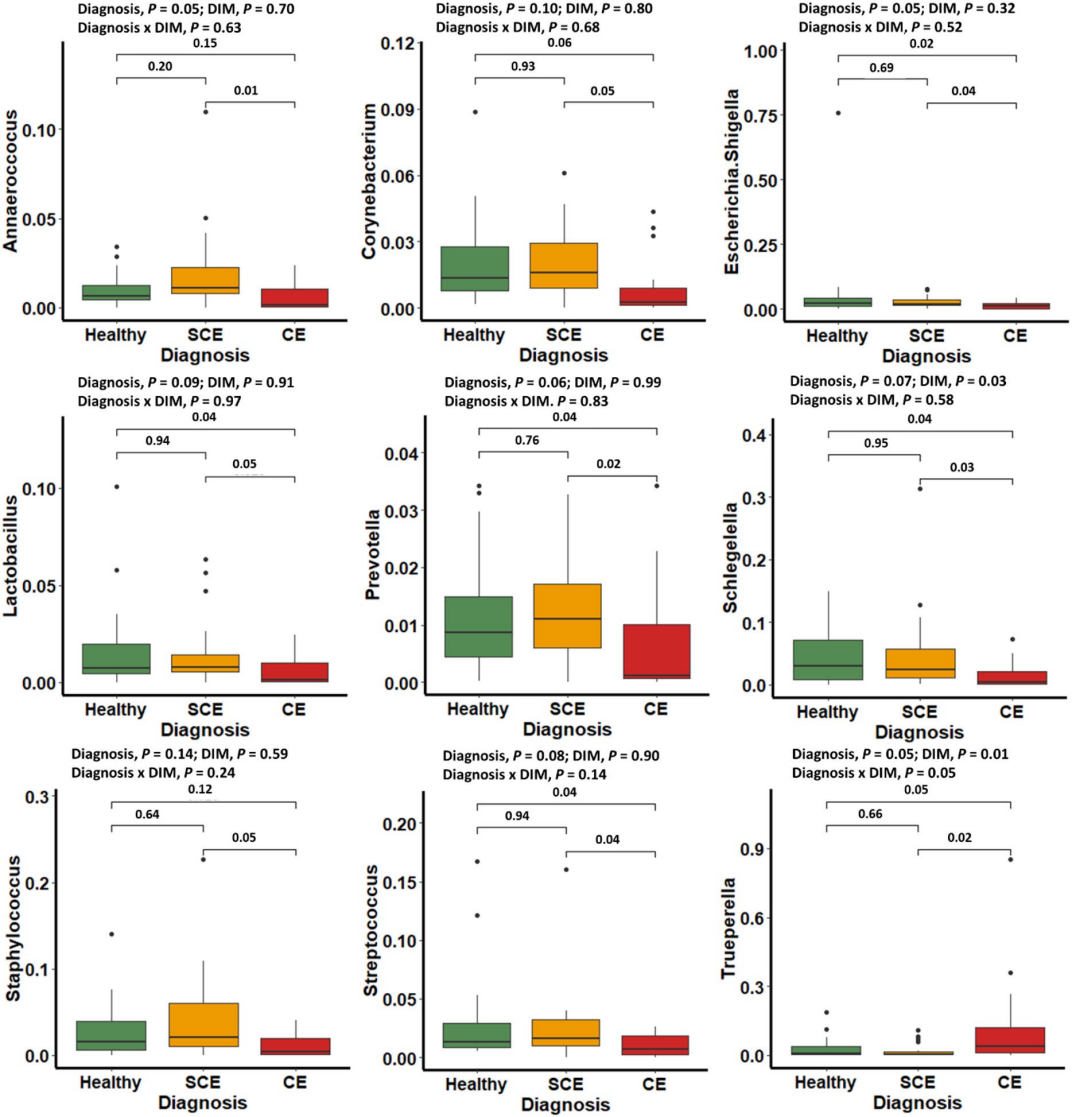
Relative abundance of the most influential bacteria genera of cows retrospectively selected based on their uterine health status in the fifth week postpartum and classified as healthy (n = 8), with subclinical endometritis (SCE; n = 8; < 50% purulent vaginal discharge and > 5% endometrial neutrophils (PMN)), or with clinical endometritis (CE; n = 5; ˃ 50% purulent vaginal discharge and > 5% endometrial PMN). No differences in relative abundance of bacteria genera was found between healthy and SCE cows (*P* > 0.20). There was lower relative abundance of *Escherichia Shigella, Lactobacillus, Prevotella, Schlegelella,* and *Streptococcus* in healthy and SCE in comparison to CE cows (*P* < 0.05). Cows with CE had greater relative abundance of *Trueperella* than healthy and SCE, and SCE had greater abundance of *Anaerococcus, Corynebacterium,* and *Staphylococcus* than CE (*P* < 0.05).

**Figure 5:**
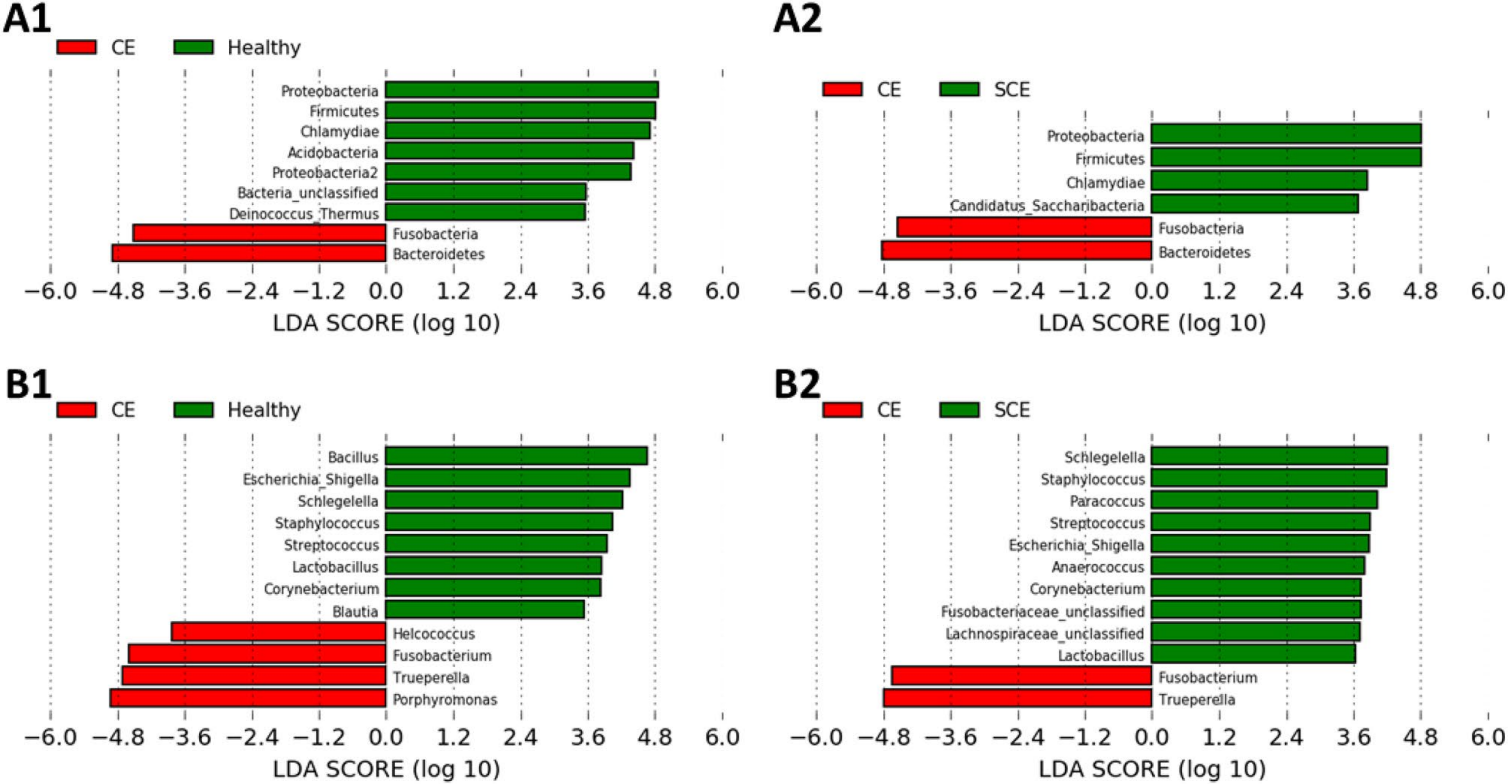
Linear discriminant analysis (LDA) effect size plots showing the differences in uterine microbiota among cows diagnosed in the fifth week postpartum as healthy (n = 8), subclinical endometritis (SCE; n = 8; < 50% purulent vaginal discharge and > 5% endometrial neutrophils (PMN)), or with clinical endometritis (CE; n = 5; ˃ 50% purulent vaginal discharge and > 5% endometrial PMN). Histograms show the LDA effect size computed for features at the phyla (**A1**, **A2**) and genera (**B1**, **B2**) levels. Enriched features for healthy and SCE are indicated with positive LDA scores (green), and enriched features in CE cows are indicated with negative LDA scores (red). Only features with *P* ˃ 0.05 and an effect size cut-off of 3.5 are plotted.

Days in milk at sampling (10 vs. 21 vs. 35) did not have an effect on alpha diversity indices (Chao1, Shannon-Weiner, or Camargo’s evenness) in cows classified as healthy, SCE, or CE (Fig. 1A). Similarly, DIM at sampling did not have an effect on beta diversity at the phylum or genera levels (Fig. 1B and C). Alpha diversity for bacteria genera and beta diversity for bacteria phyla and genera showed differences in the uterine microbiome in cows with CE compared with healthy or SCE cows (Fig. 1A–C). No differences of alpha or beta diversity were found between healthy and SCE (Fig. 1A–C). Figure 1D shows that 343 bacteria were common to all groups, but healthy and SCE cows had a greater number (n = 76) of bacteria core genera in common than CE and healthy (n = 6) or CE and SCE (n = 5). These patterns were consistent across sampling days (Supplemental Fig. S2). At the phylum level, cows with CE had lesser relative abundance of *Firmicutes* and *Proteobacteria* than healthy or SCE cows (Fig. 2). Cows with SCE had lesser relative abundance of *Bacteroidetes* than CE cows. At the genera level, cows diagnosed with CE had greater relative abundance of *Trueperella* but lower relative abundance of *Escherichia Shigella, Lactobacillus, Prevotella, Schlegelella,* and *Streptococcus* than healthy or SCE cows (Figs. 3 and 4). Cows with SCE had greater relative abundance of *Anaerococcus*, *Corynebacterium*, and *Staphylococcus* than CE cows (Fig. 4). No differences in relative abundance of bacteria genera was found between healthy and SCE cows (Fig. 4). Interestingly, the relative abundance of most bacteria phyla and genera remained stable among uterine health categories and among sampling days within healthy, SCE, or CE cows (Fig. 5).

### Linear discriminant analysis effect size

At the phylum level, *Fusobacteria* and *Bacteroidetes* were discriminately greater (LDA sores > 4) in CE in comparison to healthy and SCE cows (Fig. 5 A1 and A2). At the genus level, CE cows had discriminately greater (LDA sores > 3.5) *Helcococcus*, *Fusobacterium*, *Trueperella*, and *Porphyromonas,* and *Fusobacterium* and *Trueperella* than healthy and SCE cows, respectively (Fig. 5 B1 and B2). LDA-LEfSe did not find differences (phylum or genera levels) between healthy and SCE cows.

### Heatmap and network analysis plots

**Figure 6:**
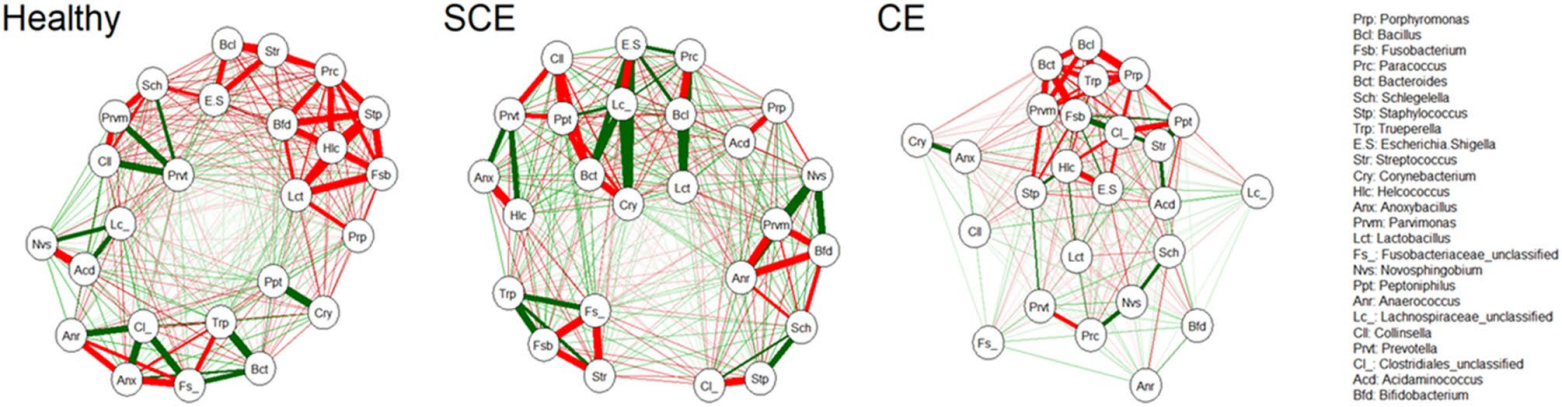
Network analysis based of the most influential uterine bacteria genera of postpartum dairy cows (n = 21) in samples collected at 10, 21, and 35 days in milk (DIM; together). Cows were retrospectively selected based on their uterine health status in the fifth week postpartum and classified as healthy (n = 8), with subclinical endometritis (SCE; n = 8; < 50% purulent vaginal discharge and > 5% endometrial neutrophils (PMN)), or with clinical endometritis (CE; n = 5; ˃ 50% purulent vaginal discharge and > 5% endometrial PMN). Green and red lines indicate positive and negative correlations, respectively. The thickness of a line is proportional to the strength of the (Spearman) correlation.

Supplemental Figure S3 and S4 illustrate fold changes of the most influential uterine bacteria phyla and genera at 10, 21, and 35 DIM in healthy, SCE, and CE cows. Heatmaps do not show strong clustering amongst DIM or uterine health status. Network analysis plots in Fig. 6 and Supplemental Fig. S5 show the correlations of the most influential bacteria genera in healthy, SCE, and CE cows. The bacterial network in CE is less dynamic (fewer bacterial interactions and weaker bacterial correlations) than for healthy and SCE cows. This trend is particularly visible in Fig. 6 with few bacterial interactions in CE cows. Among these interactions, *Trueperella*, *Bacteroides*, *Porphyromonas*, and *Bacillus* are negatively correlated. However, in healthy and SCE cows the interaction network is more complex with multiple correlations (positive and negative) among bacteria genera (Fig. 6).

### Bacterial culture and 16S rRNA gene sequencing

**Figure 7:**
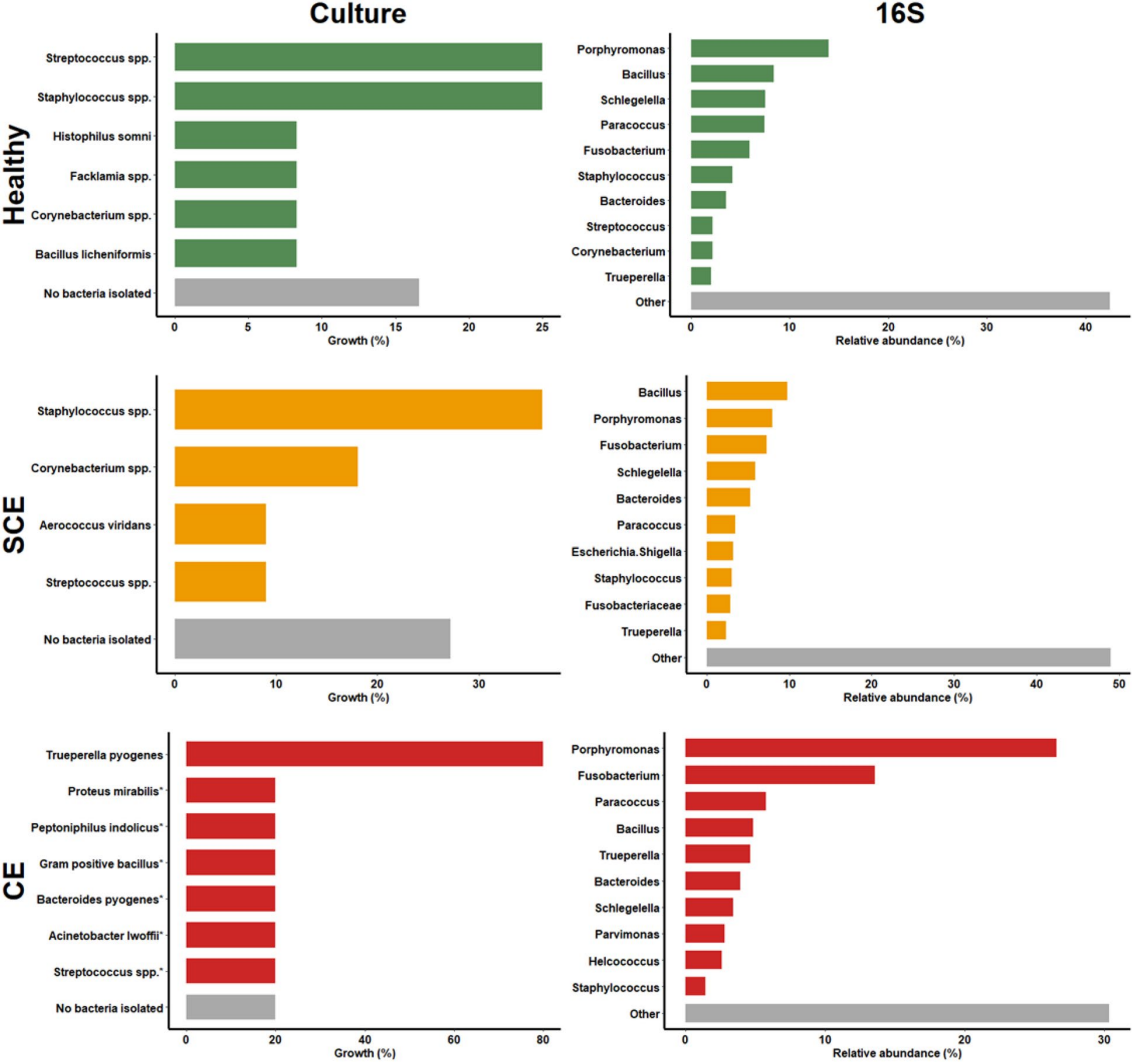
Comparison of bacteriologic results by method: growth proportion of uterine bacterial populations (isolated from bacterial culture; left hand column), and relative abundance of the top ten uterine bacteria genera (metagenomic analyses of the 16S rRNA gene sequence; right hand column) of postpartum dairy cows (n = 21) in samples collected at 35 days in milk. Cows were retrospectively selected based on their uterine health status in the fifth week postpartum and classified as healthy (n = 8), with subclinical endometritis (SCE; n = 8; < 50% purulent vaginal discharge and > 5% endometrial neutrophils (PMN)), or with clinical endometritis (CE; n = 5; ˃ 50% purulent vaginal discharge and > 5% endometrial PMN).

Figure 7 shows the proportions of uterine bacteria isolated from bacterial culture and the relative abundance of the top 10 uterine bacteria genera from metagenomic analyses in samples collected at 35 DIM and diagnosed as healthy, SCE or CE. Bacteria that grew in culture were often represented within the most abundant bacterial genera found in the metagenomic sequencing.

## Discussion

We found that the uterine microbiota of cows with SCE was similar to healthy cows, but the microbiome differed in cows with CE. At the phylum level, healthy and SCE cows had greater relative abundance of *Firmicutes* and *Proteobacteria* and lower relative abundance of *Bacteroidetes* and *Fusobacteria* than CE cows. At the genus level, cows with CE had microbiota characterized by greater relative abundance of *Fusobacterium* and *Trueperella* lower relative abundance of *Escherichia Shigella, Lactobacillus, Prevotella, Schlegelella, Staphylococcus,* and *Streptococcus* than healthy and SCE cows. The uterine bacterial composition within all three categories of uterine health generally remained stable across 10, 21, and 35 DIM. Bacteria that grew in culture had some commonalities within the most abundant bacteria found in the16S rRNA gene sequencing in samples collected simultaneously at 35 DIM. Because the microbiome of healthy cows and those with SCE was similar, our results support the notion that SCE is a consequence of dysregulation of inflammation rather than changes in uterine microbiota.

We approached our data analyses using different methods. For the extraction of the ‘most influential’ bacteria among different reproductive health status we used the *simper* function of the R package vegan^22^. The *simper* function performs pairwise comparisons of groups of sampling units and finds the average contributions of each bacteria to the average overall Bray–Curtis dissimilarity^22^. The contribution of each selected bacteria is at least 70% of the differences between groups. Thus, the function returns more information than can be accessed directly from the individual sums of the relative abundances. For each bacteria phylum and genus identified by *simper*, we performed linear regression models. However, due to our relatively small sample size (particularly for CE), linear regression tests may generate (mainly) type 2 error. Consequently, we also performed LDA-LEfSe. LDA-LEfSe uses Kruskal–Wallis tests to detect significant features, and then uses LDA to estimate the effect size of each differentially abundant feature^27^. Although LDA-LEfSe identified a slightly greater number of influential bacteria, most of the results were coincident between *simper* and LDA-LEfSe.

The uterine microbiota has been shown to be a dynamic consortium, particularly within the first week after calving and in cows that develop clinical uterine disease^12,30,31^. Santos and Bicalho^12^ observed shifts of bacterial diversity in metritic, endometritic, and healthy cows in two consecutive uterine samples collected within the first 10 DIM. However, they showed that the core bacterial community seemed to be stable for each uterine condition by the fifth week postpartum. We found similar results, with stable uterine microbiota at 10, 21, and 35 DIM within healthy, SCE, and CE cows. During this time, there was no variation in alpha or beta diversity and no major changes in the abundance of the most influential bacteria phyla or genera. Interestingly, *Trueperella* relative abundance was greater at 10 DIM than in samples collected at 21 and 35 DIM in CE cows. We expected more divergence over time between healthy and diseased cows, but consistent with other authors^12,18^, it appears that dysbiosis associated with CE is seeded within the first week postpartum and this persisted across 10, 21, and 35 DIM.

Our results from next generation sequencing of the 16S rRNA gene support earlier findings of loss of bacterial diversity in cows with CE^4,12,17^. At the phylum level, CE was characterized by greater relative abundance of *Bacteroidetes* and *Fusobacteria*, and lower relative abundance of *Firmicutes* and *Proteobacteria* (among others) than in healthy or SCE cows. However, as many other studies described^12,17^, a specific *Actinobacteria*, *Trueperella* played a major role in CE cows. However, it has been argued that the 16S rRNA gene sequences could be from nonviable remnants of bacteria associated with the uterine involution process^5^. Our data comparing culture-dependent and -independent methods partially address the debate about discrepancies between the bacterial profiles from these methods. Our culture results showed that *Trueperella pyogenes* was present in 4 of 5 CE cases. In 3 cases it was uniquely present, and in 1 case *Trueperella pyogenes* was found in a mixed bacterial infection. In the remaining CE case, no bacterial growth was found. The top two bacteria genera discovered in the 16S rRNA gene sequencing were *Fusobacterium* and *Porphyromonas*, pathogens described to act synergistically with *Trueperella* by others^4,17^. The network analysis plots interestingly show a positive correlation among some important players recognised in previous studies^17,30^, such as *Parvimonas* with *Porphyromonas*, and *Fusobacterium* with *Peptoniphilus* and *Helcococcus* at 10 DIM, *Fusobacterium* with *Trueperella*, and *Bacillus* with *Helcococcus* at 21 DIM, and *Trueperella* with *Parvimonas*, *Peptoniphilus,* and *Helcococcus* at 35 DIM. Therefore, the co-occurrence and interactions of a number uterine pathogens appear to be crucial for the development of CE. However, the interactions among bacteria and of bacteria with the host and the uterine environment require further study. It is not clear if other bacteria in the early postpartum period may help to establish the conditions for subsequent “overgrowth” of *Trueperella* by 10 DIM.

The causes of persistent of inflammation in SCE remain unclear. Our data support that known uterine pathogens are not associated with SCE. Neither alpha, beta, nor individual bacteria phyla or genera abundances differed between healthy and SCE cows, and these measures remained unchanged across sampling days. These results are in line with the findings of Wang et al*.*^17^, who concluded that common uterine pathogens were not associated with SCE at 30 DIM. They suggested that commensal bacteria could play a role in the pathogenesis of SCE. The patterns of bacterial correlations were in overall distinct but with little difference between SCE and healthy cows across sampling days. Aerobic and anaerobic culture at 35 DIM yielded mixed growth of bacteria such as *Staphylococcus*, *Streptococcus*, and *Corynebacterium* in healthy and SCE cows. Interestingly, bacteria that grew in culture were often present within the most abundant bacteria found in the16S rRNA gene sequencing. These results support that studies that failed to find an association between SCE and pathogenic bacteria were not biased by their lack of growth in culture media^15,16^, and that pathogenic bacteria are not associated with SCE. Our bacterial culture data provide additional description, but we acknowledge limitations, including a small sample size, especially for CE cows. Our culture data would have been strengthened by addition of qPCR on the colonies and by inclusion of a positive control for the anaerobic culture to increase confidence that conditions were provided for fastidious organisms. Nevertheless, these results on the whole support the hypothesis that SCE is associated with maladaptation to negative energy balance and dysregulation of inflammation rather than uterine infection as has been proposed^7,13^.

## Conclusion

The uterine microbiota was generally stable across 10, 21, and 35 DIM for healthy, SCE, and CE cows. Cows with CE had a different microbiome, with increased relative abundance of *Bacteroidetes* and *Fusobacteria* (phylum level), and *Fusobacterium* and *Trueperella* (genera level) in comparison to healthy or SCE cows. Bacteria that grew in culture were often present within the most abundant bacteria in the16S rRNA gene sequencing. Therefore, prevention or treatment of dysbiosis is a suitable direction for research to mitigate CE or PVD. Uterine bacterial composition was not different between healthy and SCE cows. This study supports the hypothesis that bacterial pathogens in the uterus are not associated with SCE, which points to the alternative hypothesis that regulation of uterine inflammation is worthy of pursuit for prevention and treatment of SCE.

## Supplementary information

Supplementary Figures

Supplementary Tables

## References

[1] Galvão KN, Bicalho RC, Jeon SJ (2019). Symposium review: the uterine microbiome associated with the development of uterine disease in dairy cows. J. Dairy Sci..

[2] Sheldon IM, Cronin JG, Bromfield JJ (2019). Tolerance and innate immunity shape the development of postpartum uterine disease and the impact of endometritis in dairy cattle. Annu. Rev. Anim. Biosci..

[3] Bicalho ML, Machado VS, Higgins CH, Lima FS, Bicalho RC (2017). Genetic and functional analysis of the bovine uterine microbiota Part I: Metritis versus healthy cows. J. Dairy Sci..

[4] Bicalho MLS, Lima S, Higgins CH, Machado VS, Lima FS (2017). Genetic and functional analysis of the bovine uterine microbiota. Part II: purulent vaginal discharge versus healthy cows. J. Dairy Sci..

[5] Moore SG, Ericsson AC, Poock SE, Melendez P, Lucy MC (2017). Hot topic: 16S rRNA gene sequencing reveals the microbiome of the virgin and pregnant bovine uterus. J. Dairy Sci..

[6] Baker JM, Chase DM, Herbst-Kralovetz M (2018). Uterine microbiota: residents, tourists, or invaders?. Front Immunol..

[7] Pascottini OB, LeBlanc SJ (2020). Modulation of immune function in the bovine uterus peripartum. Theriogenology.

[8] Dohmen MJW, Lohuis JACM, Huszenicza G, Nagy P, Gacs M (1995). The relationship between bacteriological and clinical findings in cows with subacute/chronic endometritis. Theriogenology.

[9] Drillich M, Beetz O, Pfützner A, Sabin M, Sabin HJ (2001). Evaluation of a systemic antibiotic treatment of toxic puerperal metritis in dairy cows. J. Dairy Sci..

[10] Williams EJ, Fischer DP, Pfeiffer DU, England GCW, Noakes DE (2005). Clinical evaluation of postpartum vaginal mucus reflects uterine bacterial infection and the immune response in cattle. Theriogenology.

[11] Dubuc J, Duffield TF, Leslie KE, Walton JS, LeBlanc SJ (2010). Definitions and diagnosis of postpartum endometritis in dairy cows. J. Dairy Sci..

[12] Santos TMA, Bicalho RC (2012). Diversity and succession of bacterial communities in the uterine fluid of postpartum metritic, endometritic and healthy dairy cows. PLoS ONE.

[13] Wagener K, Gabler C, Drillich M (2017). A review of the ongoing discussion about definition, diagnosis and pathomechanism of subclinical endometritis in dairy cows. Theriogenology.

[14] Sens A, Heuwieser W (2013). Presence of *Escherichia coli, Trueperella pyogenes*, a-hemolytic streptococci, and coagulase-negative staphylococci and prevalence of subclinical endometritis. J. Dairy Sci..

[15] Prunner I, Wagener K, Pothmann H, Ehling-Schulz M, Drillich M (2014). Risk factors for uterine diseases on small- and medium-sized dairy farms determined by clinical, bacteriological, and cytological examinations. Theriogenology.

[16] Madoz LV, Giuliodori MJ, Migliorisi AL, Jaureguiberry M, de la Sota RL (2014). Endometrial cytology, biopsy, and bacteriology for the diagnosis of subclinical endometritis in grazing dairy cows. J. Dairy Sci..

[17] Wang ML, Liu MC, Xu J, An LG, Wang JF (2018). Uterine microbiota of dairy cows with clinical and subclinical endometritis. Front. Microbiol..

[18] Galvão KN, Higgins CH, Zinicola M, Jeon SJ, Korzec H (2019). Effect of pegbovigrastim administration on the microbiome found in the vagina of cows postpartum. J. Dairy Sci..

[19] Pascottini OB, Van Schyndel SJ, Spricigo JFW, Carvalho MR, Mion B (2020). Effect of anti-inflammatory treatment on systemic inflammation, immune function, and endometrial health in postpartum dairy cows. Sci Rep..

[20] Pascottini OB, Spricigo JFW, Van Schyndel SJ, Mion B, Rousseau J (2020). Effects of parity, blood progesterone, and non-steroidal anti-inflammatory treatment on the dynamics of the uterine microbiota of healthy postpartum dairy cows. bioRxiv.

[21] Walters W, Hyde ER, Berg-Lyons D, Ackermann G, Humphrey G (2015). Improved bacterial 16S rRNA (V4 and V4–5) and fungal internal transcribed spacer marker gene primers for microbial community surveys. mSystems.

[22] Oksanen, J., Kindt, R., Legendre, P. & O'Hara, B. *Vegan: Community Ecology Package. R package version 1.15–4*. https://CRAN.R-project.org/package=vegan (2011).

[23] Vavrek MJ (2011). Fossil: palaeoecological and palaeogeographical analysis tools. Palaeontol. Electron..

[24] McMurdie PJ, Susan H (2013). Phyloseq: an R package for reproducible interactive analysis and graphics of microbiome census data. PLoS ONE.

[25] Davis NM, Proctor DM, Holmes SP, Relman DA, Callahan BJ (2018). Simple statistical identification and removal of contaminant sequences in marker-gene and metagenomics data. Microbiome.

[26] Bah T (2011). Inkscape: Guide to a Vector Drawing Program.

[27] Segata N, Izard J, Waldron L, Gevers D, Miropolsky L (2011). Metagenomic biomarker discovery and explanation. Genome Biol..

[28] Day, A. *heatmap.plus: Heatmap with more Sensible Behavior*. https://CRAN.R-project.org/package=heatmap.plus (2012).

[29] Epskamp S, Cramer AOJ, Waldorp LJ, Schmittmann VD, Borsboom D (2012). qgraph: Network visualizations of relationships in psychometric data. J. Stat. Softw..

[30] Jeon SJ, Vieira-Neto A, Gobikrushanth M, Daetz R, Mingoti RD (2015). Uterine microbiota progression from calving until establishment of metritis in dairy cows. Appl. Environ. Microbiol..

[31] Miranda-CasoLuengo R, Lu J, Williams EJ, Miranda-CasoLuengo AA, Carrington SD (2019). Delayed differentiation of vaginal and uterine microbiomes in dairy cows developing postpartum endometritis. PLoS ONE.

