# Supplementary material for: Dynamics of uterine microbiota in postpartum dairy cows with clinical or subclinical endometritis
Source: Sci Rep. 2020 Jul 23;10:12353. doi: 10.1038/s41598-020-69317-z (PMC7378066; doi:10.1038/s41598-020-69317-z)
Supplement: Supplementary file 1 — Supplementary Figures [file 41598_2020_69317_MOESM1_ESM.docx]

**Dynamics of uterine microbiota in postpartum dairy cows with clinical or subclinical endometritis**

O. Bogado Pascottini^1*^, S. J. Van Schyndel^1^, J. F. W. Spricigo^2^, J. Rousseau^3^, J. S. Weese^3^, S. J. LeBlanc^1^

*^1^Population Medicine, Ontario Veterinary College, University of Guelph, Guelph, ON N1G 2W1, Canada.*

*^2^Department of Animal Biosciences, University of Guelph, Guelph, ON N1G 2W1, Canada.*

*^3^Department of Pathobiology, Ontario Veterinary College, University of Guelph, Guelph, ON N1G 2W1, Canada*.

**^*^Corresponding author:** O. Bogado Pascottini; [osvaldo.bogado@ugent.be](mailto:osvaldo.bogado@ugent.be)


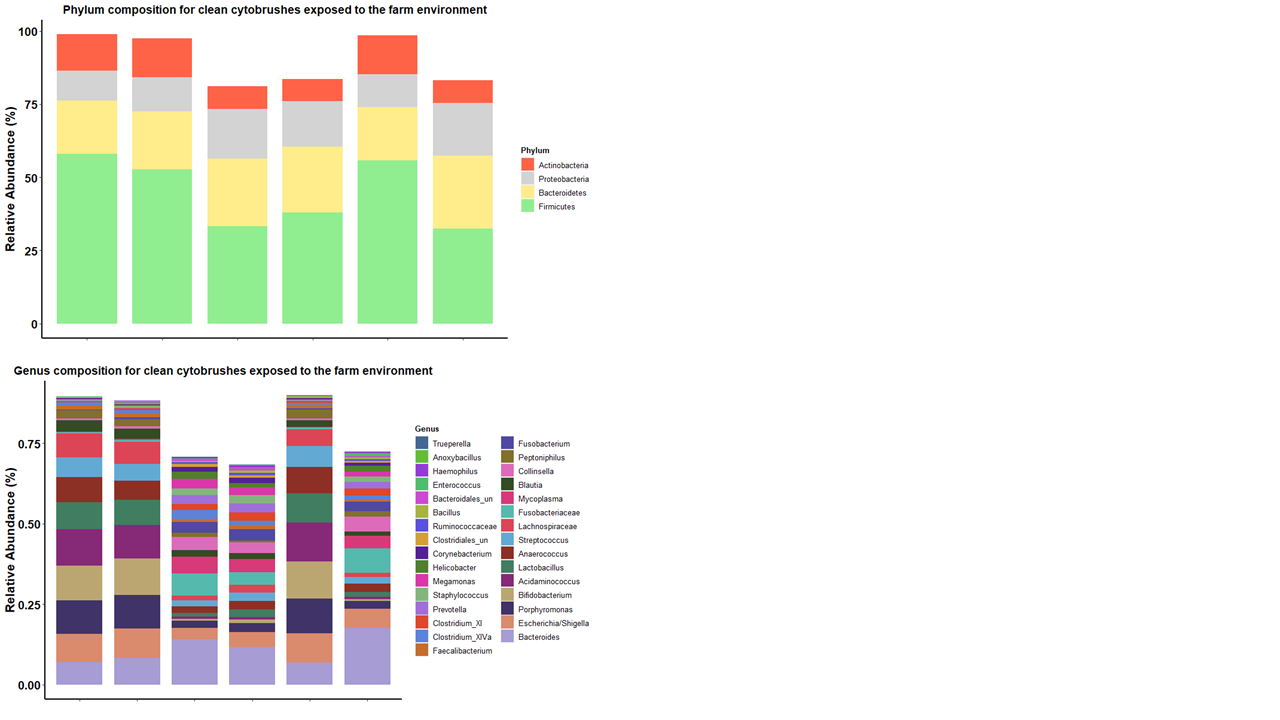


**Supplemental Figure S1.** Relative abundance of bacterial phyla and genera of six sterile cytobrushes exposed to the air in the barn as during the experimental sampling from cows. These cytobrush samples were used for decontamination of the metagenomics data.


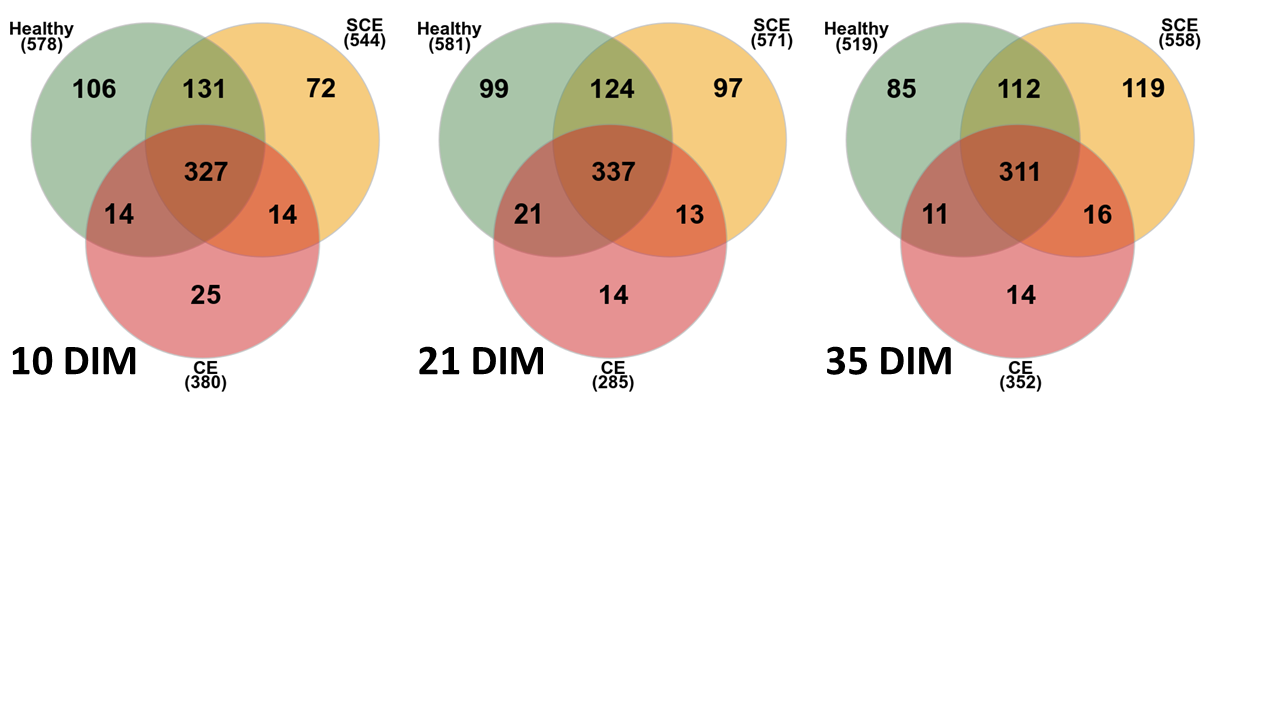


**Supplemental Figure S2.** Uterine bacterial composition of postpartum dairy cows (n = 21) in samples collected at 10, 21, and 35 d in milk (DIM). Cows were retrospectively selected based on their uterine health status in the fifth week postpartum and classified as healthy (n = 8), with subclinical endometritis (SCE; n = 8; < 50% purulent vaginal discharge and > 5% endometrial neutrophils (PMN)), or with clinical endometritis (CE; n = 5; ˃ 50% purulent vaginal discharge and > 5% endometrial PMN). Venn diagrams showing that healthy and SCE shared greater numbers of core bacteria genera than either group did with cows with CE at 10, 21, and 35 DIM.

**
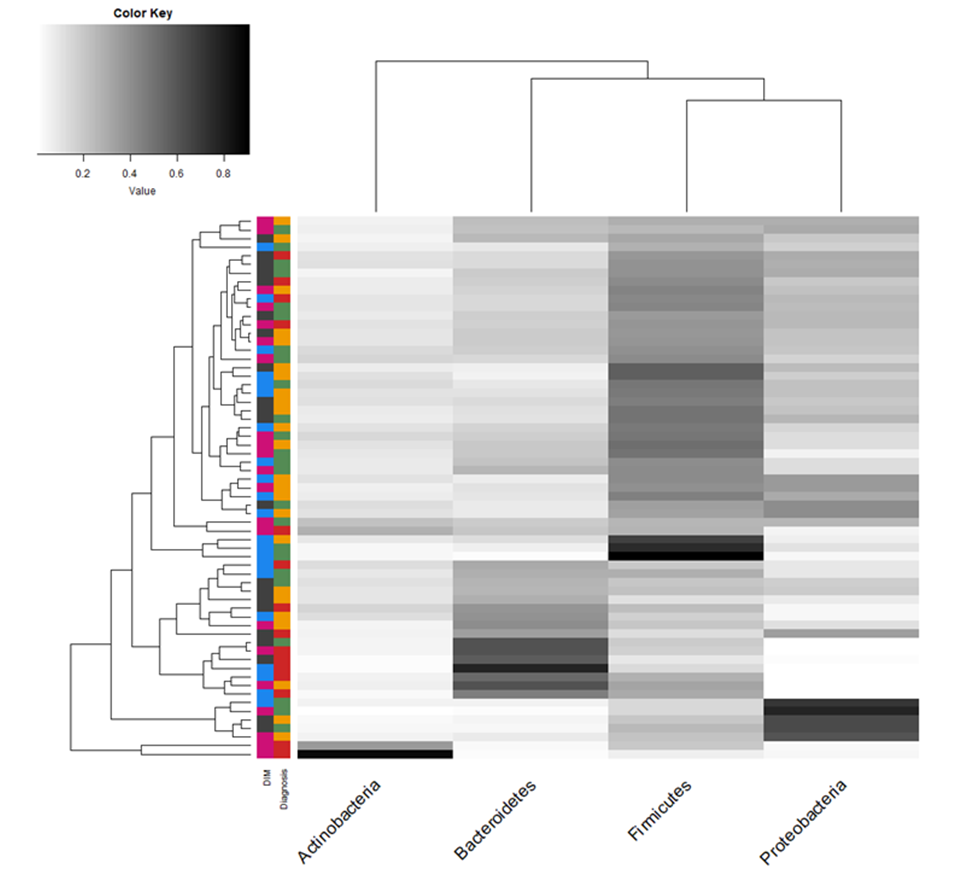
**

**Supplemental Figure S3.** Heatmap with average linkage clustering based on Bray–Curtis distance, showing the relative abundances of the most influential uterine bacteria phyla of postpartum dairy cows (n = 21) in samples collected at 10, 21, and 35 d in milk (DIM). Cows were retrospectively selected based on their uterine health status in the fifth week postpartum and classified as healthy (n = 8), with subclinical endometritis (SCE; n = 8; < 50% purulent vaginal discharge and > 5% endometrial neutrophils (PMN)), or with clinical endometritis (CE; n = 5; ˃ 50% purulent vaginal discharge and > 5% endometrial PMN). The relative abundance is indicated by a gradient of color from black (high abundance) to white (low abundance). Similarity dendrogram shows the unweighted pair group method with arithmetic mean (UPGMA) clustering of bacterial phyla by DIM (pink for 10 DIM, light blue for 21 DIM, and dark grey for 35 DIM), and uterine disease diagnosis (green for healthy, orange for SCE, and red for CE). The cophenetic correlation coefficients were 0.89 for 10 DIM, 0.86 for 21 DIM, and 0.85 for 35 DIM, and 0.88 for healthy, 0.87 for SCE, and 0.89 for CE.

**
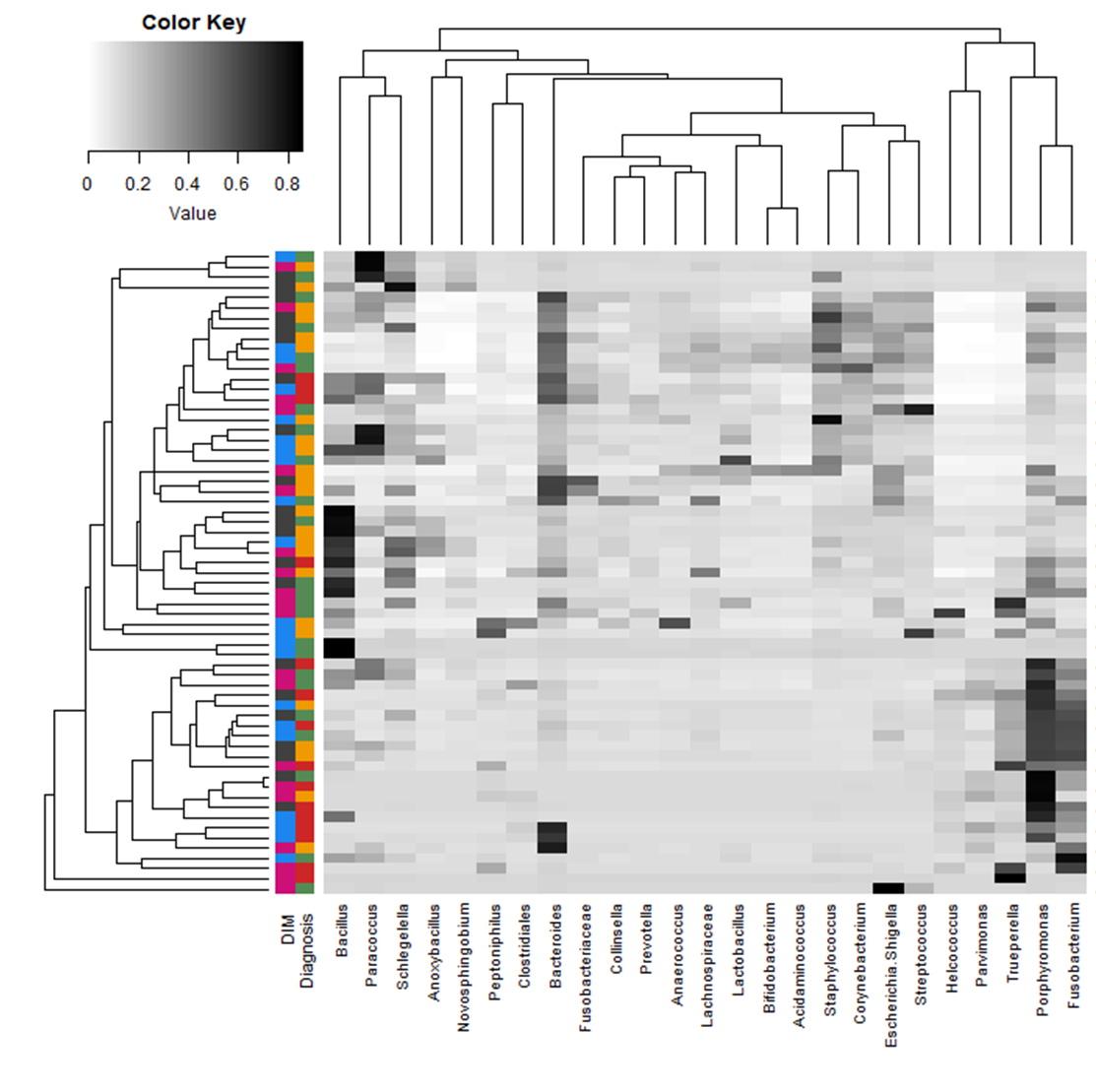
**

**Supplemental Figure S4.** Heatmap with average linkage clustering based on Bray–Curtis distance showing the relative abundances of the most influential uterine bacteria genera of postpartum dairy cows (n = 21) in samples collected at 10, 21, and 35 days in milk (DIM). Cows were retrospectively selected based on their uterine health status in the fifth week postpartum and classified as healthy (n = 8), with subclinical endometritis (SCE; n = 8; < 50% purulent vaginal discharge and > 5% endometrial neutrophils (PMN)), or with clinical endometritis (CE; n = 5; ˃ 50% purulent vaginal discharge and > 5% endometrial PMN). The relative abundance is indicated by a gradient of color from black (high abundance) to white (low abundance). Similarity dendrogram shows the unweighted pair group method with arithmetic mean (UPGMA) clustering of bacterial genera by DIM (pink for 10 DIM, light blue for 21 DIM, and dark grey for 35 DIM), and uterine disease diagnosis (green for healthy, orange for SCE, and red for CE). The cophenetic correlation coefficients were 0.77 for 10 DIM, 0.84 for 21 DIM, and 0.9 for 35 DIM, and 0.85 for healthy, 0.84 for SCE, and 0.86 for CE.


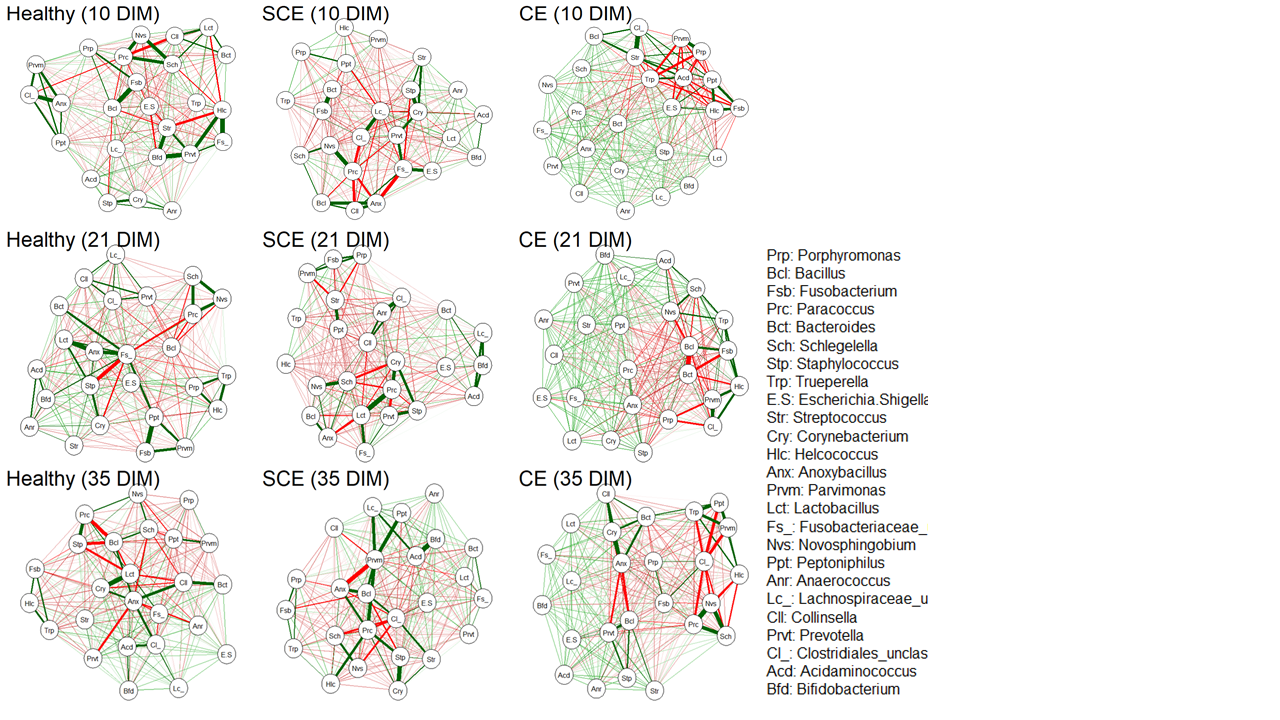


**Supplemental Figure S5.** Network analysis based of the most influential uterine bacteria genera of postpartum dairy cows (n = 21) in samples collected at 10, 21, and 35 days in milk (DIM). Cows were retrospectively selected based on their uterine health status in the fifth week postpartum and classified as healthy (n = 8), with subclinical endometritis (SCE; n = 8; < 50% purulent vaginal discharge and > 5% endometrial neutrophils (PMN)), or with clinical endometritis (CE; n = 5; ˃ 50% purulent vaginal discharge and > 5% endometrial PMN). Green and red lines indicate positive and negative correlations, respectively. The thickness of line is proportional to the strength of the (Spearman) correlation.
