# Supplementary material for: Dynamics of uterine microbiota in postpartum dairy cows with clinical or subclinical endometritis
Source: Sci Rep. 2020 Jul 23;10:12353. doi: 10.1038/s41598-020-69317-z (PMC7378066; doi:10.1038/s41598-020-69317-z)
Supplement: Supplementary file 2 — Supplementary Tables [file 41598_2020_69317_MOESM2_ESM.docx]

**Dynamics of uterine microbiota in postpartum dairy cows with clinical or subclinical endometritis**

O. Bogado Pascottini^1*^, S. J. Van Schyndel^1^, J. F. W. Spricigo^2^, J. Rousseau^3^, J. S. Weese^3^, S. J. LeBlanc^1^

*^1^Population Medicine, Ontario Veterinary College, University of Guelph, Guelph, ON N1G 2W1, Canada.*

*^2^Department of Animal Biosciences, University of Guelph, Guelph, ON N1G 2W1, Canada.*

*^3^Department of Pathobiology, Ontario Veterinary College, University of Guelph, Guelph, ON N1G 2W1, Canada*.

**^*^Corresponding author:** O. Bogado Pascottini; [osvaldo.bogado@ugent.be](mailto:osvaldo.bogado@ugent.be)

| **Supplementary Table S1.** Descriptive data on the vaginal discharge (VD) score, endometrial polymorphonuclear neutrophil percentage (PMN%), and uterine health status of postpartum dairy cows (n = 21) in samples collected at 35 days in milk (DIM). Cows were classified based on their uterine health status as healthy, with SCE (< 50% purulent vaginal discharge and > 5% PMN), or with clinical endometritis (CE; ˃ 50% PVD and > 5% PMN). From the same uterine sample, bacteria were isolated using aerobic and anaerobic bacterial culture and sequenced using the 16S rRNA gene (metagenomics). | | | | | | | |
| --- | --- | --- | --- | --- | --- | --- | --- |
| **Cow ID** | **VD score** | **PMN%** | **Diagnosis** | **Culture** | | **Microbiome (16S)** | |
|  |  |  |  | **Bacteria** | **Growth^1^** | **Relative abundance** | **Ranking^2^** |
| 3970 | 1 | 0 | Healthy | No pathogens isolated | None | None | None |
| 4381 | 1 | 0 | Healthy | Histophilus somni  Streptococcus pluranimalium  Facklamia hominis  Corynebacterium rénale | +  +  +  + | 0.001366  0.053112  0.000223  0.048365 | 84  4  93  5 |
| 4507 | 0 | 0 | Healthy | Staphylococcus xylosus | ++ | 0.14019 | 3 |
| 4517 | 0 | 2 | Healthy | No pathogens isolated | None | None | None |
| 4626 | 0 | 1 | Healthy | Staphylococcus equorum  Staphylococcus epidermis | +  + | 0.003595 | 34  34 |
| 4629 | 0 | 1 | Healthy | No pathogens isolated |  | None | None |
| 4630 | 0 | 2 | Healthy | Bacillus licheniformis  Streptococcus pluranimalium | +  + | 0.240113  0.020921 | 1  9 |
| 4654 | 0 | 2 | Healthy | Streptococcus thoraltensis  Staphylococcus chromogenes | +  + | 0.0083  0.000176 | 9  19 |
| 4077 | 0 | 18 | SCE | Staphylococcus hyicus  Aerococcus viridans  Staphylococcus agnetis  Corynebacterium spp. | +  +  +  + | 0.108915  0.001093  0.060993 | 1  101  1  3 |
| 4310 | 0 | 50 | SCE | No pathogens isolated | + | None | None |
| 4406 | 0 | 24 | SCE | Corynebacterium spp.  Staphylococcus xylosus | +  + | 0.015868  0.022566 | 12  6 |
| 4523 | 0 | 69 | SCE | No pathogens isolated |  | None | None |
| 4557 | 0 | 13 | SCE | Staphylococcus auricularis  Staphylococcus xylosus | +  + | 0.008909 | 13 |
| 4642 | 0 | 17 | SCE | No pathogens isolated |  | None | None |
| 4644 | 0 | 22 | SCE | Staphylococcus xylosus  Staphylococcus equorum | +  + | 0.004383 | 38  38 |
| 4675 | 1 | 25 | SCE | Streptococcus pluranimalium | + | 0.028624 | 5 |
| 4371 | 2 | 35 | CE | Trueperella pyogenes | ++++ | 0.008198 | 7 |
| 4481 | 3 | 10 | CE | No pathogens isolated |  | None | None |
| 4628 | 2 | 13 | CE | Trueperella pyogenes | +++ | 0.038809 | 5 |
| 4648 | 2 | 34 | CE | Trueperella pyogenes  Streptococcus alpha hemolytic  Streptococcus pluranimalium  Acinetobacter lwoffii  Bacteroides pyogenes  Gram negative bacillus  Gram positive bacillus  Peptoniphilus indolicus  Proteus mirabilis | ++  ++  ++  +  +  +  +  +  + | 0.028241  0.02153  0.02153  0.002753  0.158339  0.049408  0.049408  0.005239  0.000075 | 7  12  12  60  1  4  4  37  220 |
| 4655 | 3 | 44 | CE | Trueperella pyogenes | ++++ | 0.15417 | 3 |
| ^1^4+ = large, 3+ = moderate, 2+ = few, 1+ = occasional organisms isolated.  ^2^Relative abundance ranking (16S rRNA gene) of the same bacteria genera isolated in the bacterial culture. | | | | | | | |
